# Identification of ***b***-/***y***-ions in MS/MS spectra using a two stage neural network

**DOI:** 10.1186/1477-5956-11-S1-S4

**Published:** 2013-11-07

**Authors:** James P Cleveland, John R Rose

**Affiliations:** 1 Department of Computer Science, University of South Carolina, Columbia, SC, USA

## Abstract

Independent of the approach used, the ability to correctly interpret tandem MS data depends on the quality of the original spectra. Even in the case of the highest quality spectra, the majority of spectral peaks can not be reliably interpreted. The accuracy of sequencing algorithms can be improved by filtering out such 'noise' peaks. Preprocessing MS/MS spectra to select informative ion peaks increases accuracy and reduces the processing time. Intuitively, the mix of informative versus non-informative peaks has a direct effect on the quality and size of the resulting candidate peptide search space. As the number of selected peaks increases, the corresponding search space increases exponentially. If we select too few peaks then the ion-ladder interpretation of the spectrum will contain gaps that can only be explained by permutations of combinations of amino acids. This will result in a larger candidate peptide search space and poorer quality candidates. The dependency that peptide sequencing accuracy has on an initial peak selection regime makes this preprocessing step a crucial facet of any approach, whether de novo or not, to MS/MS spectra interpretation.

We have developed a novel approach to address this problem. Our approach uses a staged neural network to model ion fragmentation patterns and estimate the posterior probability of each ion type. Our method improves upon other preprocessing techniques and shows a significant reduction in the search space for candidate peptides without sacrificing candidate peptide quality.

## Introduction

The leading tool for identifying and characterizing proteins in high-throughput proteomics is mass spectrometry. In the case of peptide sequencing, tandem mass spectrometry is the primary tool. When sequence databases do not contain the relevant proteins, we cannot use comparison methods to match experimental spectra to theoretical spectra predicted for sequences in the database. In this case we are constrained to use a de novo approach to determining the sequence of amino acids in the peptide. This requires that we sequence the peptide using only information contained in the MS/MS spectrum. We can roughly divide de novo peptide sequencing approaches into three steps: peak selection/classification, which is generally treated as a preprocessing step; generation of de novo candidate peptides; and scoring/reranking of candidate peptides. The focus of this research is peak selection. The objective of this step is to identify informative peaks in the spectrum. We are particulary interested in peaks that represent *b*-/*y*-ions. An ideal MS/MS spectrum would be free of noise and contain all of the prefix (N-terminal *b*-ion) and suffix (C-terminal *y*-ion) fragments. In practice, the MS/MS spectrum contains a complex mixture of peptide fragments and uninterpretable 'noise' peaks. Different ion types such as *a*-, *c*-, *x*-, or *z*-ions may be produced if the peptide is not cleaved at an amide bond. Internal fragments, which occur when an ion undergoes a second or third fragmentation, may also be present in the spectrum. The *b*-/*y*-ions in an ideally fragmented peptide will form two *ladders*. In the case of *b*-ions, the ladder refers to the peaks corresponding to the prefix ions observed sequentially in the spectrum (*b*_1_-ion, *b*_2_-ion, ..., *b_n_*-ion) with each prefix offset from the previous by the mass of an amino acid. Likewise, for the *y*-ion ladder we expect to see suffix ions sequentially in the spectrum (*y*_1_-ion, *y*_2_-ion, ..., *y_n_*-ion). We can use this knowledge to derive the peptide sequence. By concatenating the amino acids corresponding to the sequential mass differences in the *b*-ion ladder we construct the peptide sequence. Likewise, by concatenating the amino acids corresponding to the sequential mass differences in the *y*-ion ladder we construct the reverse peptide sequence. Since we prefer to sequence the peptide using a *b*-/*y*-ion ladder the objective of this initial step is to select the 'signal' peaks that likely represent to these ions in the spectrum.

The peaks that are selected are used in the candidate generation phase to propose peptides that are consitent with the MS/MS spectrum. Finally, the candidate peptides are scored and ranked, and the top ranking peptide is taken as the the peptide that likely generated the spectrum.

In general, other de novo approaches rely on peak intensity alone for initial filtering of the spectrum. PepNovo uses a sliding window that spans a range of 56 Da. It retains the top three peaks in ranked by intensity in each window [1]. ms2preproc uses the same sliding window approach, in addition to other intensity based methods [2]. MSNovo also uses a sliding window for peak selection. It selects the 6 most intense peaks in each 100 Da window [3]. In contrast, PILOT treats the whole spectrum as a single window and selects the top 125 most intense peaks [4]. pNovo similarly treats the entire sepctrum as a single window and selects the 100 most intense peaks [5]. This work improves upon our previous ion classifier [6]. In particular, we improved our modeling of the principal isotope and complementary peak features, described in greater detail below. We also demonstrate the effectiveness of our approach on a much larger dataset, and provide additional analysis.

Our research shows that reliance solely on relative intensity-based filtering can discard a significant number of the *b*-ions in the spectrum. We were able to outperform basic filtering techniques by incorporating peptide fragmentation features in a model for classifying peaks by ion-type. The Staged Neural Network (SNN) approach described below implements a predictive model that classifies peaks without requiring a comprehensive elaboration of peptide fragmentation processes. We demonstrate an ability to select peaks more accurately than other common approaches to peak filtering/preprocessing. This is important because better peak selection yields equally good (or better) candidate peptides in the search space while constraining the search space.

It is important to strike a good balance between precision and recall. An increase in recall will support the generation of more accurate candidates. Holding recall fixed and increasing precision will result in a reduced search space while at the same time retaining the best candidates. If recall is lowered there will have missing peaks in the ion ladder. This will result in a much larger candidate space since it will be necessary to consider all permutations of residue sets that have a mass that matches these gaps. If we lower the precision our search space will grow since more candidate peptides will be consistent with ion ladders generated from spurious peaks in the spectrum. This can make the problem intractable depending on the specific implementation of the candidate generation step. Thus, it is obvious that any de novo algorithm will benefit from an increase precision and recall in the initial *b*-/*y*-ion selection step.

## Methods

Our experiments were run on a comprehensive full factorial LC-MS/MS benchmark dataset [7]. The dataset consists of 59 LC-MS/MS analyses, in Mascot generic peak list format, of 50 protein samples extracted individually from Escherichia coli K12, yielding a total of 482 604 spectra. The dataset was filtered for doubly charged peptides ranging from 8 to 20 residues. The dataset (**D**) consists of 59 separate analyses $\left(D=\cup_{i=1}^{59}D_{i}\right)$. The scans in each *Di *were randomly divided into a training set $\left(D_{i}^{T}\right)$ and an evaluation set $\left(D_{i}^{E}\right)$. Each $D_{i}^{T}$ was trained separately, and each $D_{i}^{E}$ was classified using the classifier yielded by $D_{i}^{T}$. The classified scans in each $D_{i}^{E}$ were then combined $\left(D^{E}=\cup_{i=1}^{59}D_{i}^{E}\right)$ for calculating statistics. The same scans in **D***^E ^*were used to compute statistics for PepNovo+, pNovo, and ms2preproc. The results of this comparison are presented below.

**Figure 1:**
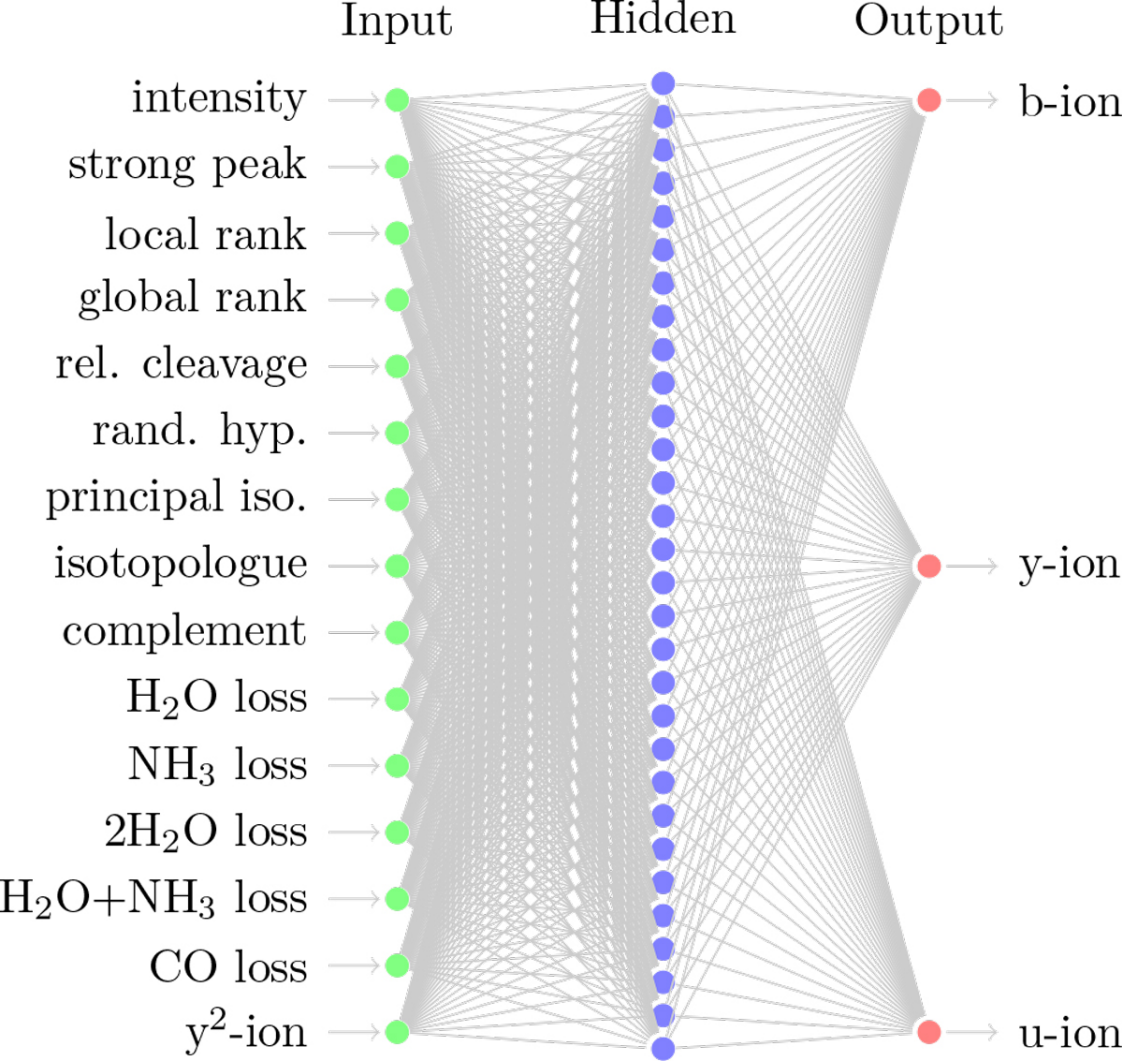
**Topology of *net*_1_**.

In an initial preprocessing step we remove peaks that have an intensity lower than 50, which was empirically determined to remove roughly half of all of the noise in the spectrum, while only removing a small number of potential *b*-/*y*-ions. This initial filtering sped up the neural network training without effecting performance or accuracy. Before training the neural network we filter the training dataset $D_{i}^{T}$ based on each peak's known ion type. For each peak in $D_{i}^{T}$ we create a target vector by labeling the peak with its ion type, either *b*-ion (1,0,0), *y*-ion (0,1,0), or *u*-ion (unknown ion) (0,0,1), each of which is a binary vector of length three. A virtual spectrum is constructed based on known CID fragmentation [8,9] of doubly charged peptides, giving the expected *b*-/*y*-ion masses for the peptide. A peak within 0.2 Da of the expected mass for a *b*-/*y*-ion is labeled accordingly and assumed to be ground truth. For each of the peak in *D_i_* we generate a feature vector (training instance) that is fed to the input layer of the neural network for training and classification. The topology of the first neural network is shown in Figure 1. We describe the features in Table 1 and the following section. $D_{i}^{T}$ is randomized and divided so that 95% of the spectra makes up the backpropagation dataset $\left(D_{i}^{T_{B}}\right)$ and 5% makes up the validation dataset (stopping criteria) $\left(D_{i}^{TV}\right)$. The backpropogagion dataset $D_{i}^{T_{B}}$ is filtered again to yeild an equivalent count of *b*, *y*, and *u *ions. Note that if we vary the ratio of *b*-/*y*-ions to *u*-ions in the training dataset we can manipulate the precision and recall of our ion classifier. For example, more *u *ions will result in a higher precision and a lower recall.

**Table 1:** Pattern features for *net*_1 _and *net*_2_

*net*_1 _pattern features		*net*_2 _pattern features	
**feature**	**value**	**feature**	**value**

intensity	N, D	intensity	N, D
strong peak	B	strong peak	B
local intensity rank	N	local intensity rank	N
global intensity rank	N	global intensity rank	N
relative cleavage position	N, D	relative cleavage position	N, D
random peak hypothesis	P	random peak hypothesis	P
principal isotope	H	principal isotope	H
isotopologue	B	isotopologue	B
complement	N	complement	${\text{P}}_{{net}_{1}}$
H_2_O neutral loss	N, D	H_2_O neutral loss	N, D
NH_3 _neutral loss	N, D	NH_3 _neutral loss	N, D
H_2_O-H_2_O neutral loss	N, D	H_2_O-H_2_O neutral loss	N, D
H_2_O-NH_3 _neutral loss	N, D	H_2_O-NH_3 _neutral loss	N, D
CO neutral loss (*a*-ion)	N, D	CO neutral loss (*a*-ion)	N, D
		N-term flanking ion	${\text{P}}_{{net}_{1}}$
		C-term flanking ion	${\text{P}}_{{net}_{1}}$

N specifies a normalized quantity, D specifies a discretized quantity, B indicates a binary value, H indicates a histogram value,  N indicates a value sampled from a normal distribution, and P indicates a probability. All peaks are processed first by *net*_1 _and and then by *net*_2_. *net*_2 _features are influenced by the preceding characterization of peaks by *net*_1_.

As part of the neural network training protocol we define an objective error function. The output layer (**o**) of the neural network corresponds to a posterior probability estimate that the input training instance is a member of the respective class defined by the target vector (**t**). It has been shown that the appropriate objective error function for such an interpretation of the output layer is the cross entropy error function [10].

$$\text{network}\;\text{error}=-\overset{2}{\underset{i=0}{\sum }}[t_{i}log(o_{i})+(1-t_{i}\text{)}log(1-o_{i})]$$

The neural network is trained using the training instances in the backpropagation training set $\left(D^{T_{B}}\right)$ repeatedly-for several epochs-until the classification performance stops improving. To achieve this, after each training epoch we classify the peaks in $D^{T_{V}}$. Once the classification error for $D^{T_{V}}$ increases compared to the previous epoch we stop training the network.

Our classifier uses two neural networks to classify peaks in a spectrum. This architecture resembles a staged neural network. Each of the neural networks is trained as described above, with differing construction of the feature vector. The structure of each neural network consists of an input layer, a single hidden layer, and an output layer. The dimension of the input layer is identical to the number of features in the training instance. The hidden layer has roughly twice as many nodes as the input layer. The output layer has three nodes that correspond to the three ion type classes. Algorithm 1 gives an overview of the training and classification protocol. In the first neural network, *net*_1_, a peak's feature vector is computed using data in the spectrum alone as described below and in Table 1. In the second neural network, *net*_2_, the classification results yielded by *net*_1 _for each peak are leveraged as additional and modified features in *net*_2_. The complementary ion feature in *net*_2 _differs from the same feature in *net*_1 _by replacing the normalized relative intensity of the complementary peak with the maximum of the *b*-/*y*-ion probability estimates in the output from *net*_1 _for the complementary peak. In other words, a hypothesised complementary peak, for which *net*_1 _has yielded a high probability of being a *b*-/*y*-ion, should serve as reinforced positive evidence that the current peak is also a *b*-/*y*-ion. We also add two new features to the *net*_2 _feature vector corresponding to flanking residues on the N and C terminal sides of the current peak. The justification for these 'leveraged' features is due to the fact that the correct peptide forms an ion ladder. Hereafter we use the term "current peak" to refer the peak for which the feature vector is being computed. The presence of flanking residues or complementary peaks serves as positive evidence that the current peak is part of an ion ladder. Our experiments demonstrate that using the outputs from *net*_1 _to modify and add features to to *net*_2 _results in higher recall compared to only using *net*_1_.

**Algorithm 1 **Staged Neural Network Training and Classification

  $net_{1}\leftarrow train\left(D^{T_{B}},\;D^{T_{V}}\right)$

  *D^T ^← classify*(*D^T^, net*_1_) {assigns b-/y-/u-ion probability estimates for peaks in *D_T_*}

  $net_{2}\leftarrow train\left(D^{T_{B}},\;D^{T_{V}}\right)$

  $D^{E}\leftarrow classify\left(D^{E}\text{, }net_{\text{1}}\right)$

  $D^{E}\leftarrow classify\left(D^{E}\text{, }net_{\text{2}}\right)$

### Description of features

The features listed in Table 1 take advantage of known fragmentation characteristics of ions produced by CID peptide fragmentation. In the following exposition, let *pI *denote the parent ion mass, and let $\vec{I}=\left\langle I_{0},I_{1},\ldots \left(I_{k}\right\rangle \right)$ denote a spectrum. For peak *Ii*, the ion mass is defined as *x_i _*= *mass*(*I_i_*), and the ion intensity/abundance is defined as *y_i _*= *intensity*(*I_i_*).

The intensity feature is computed by normalizing and then discretizing the relative peak intensity of the current peak *Ii*. Given *n *discrete intensity bins, the normalized discretized feature is defined as

$$F_{intensity}\left(I_{i}\right)=\left\lfloor n\left(y_{i}/y_{max}\right)\right\rfloor /n$$

where the *y_max _*is the most intense peak in the spectrum, and *y_i_/y_max _*is the normalized intensity for *I_i_*.

The strong peak feature is a binary value that indicates whether or not the current peak is considered 'strong' within a window around the current peak. To be called a strong peak the current peak must be one of the three most intense peaks within a window of 56 Da around the current peak. The optimal window width and the number of peaks to label as strong within the window were empirically determined.

The local and global intensity ranks are computed by normalizing the intensity rank of the current peak within a window around the current peak, or globally (the complete spectrum). The intensity-based features described so far are useful since *b*-/*y*-ions tend to have greater intensity when compared to unknown ion types.

The relative cleavage position is a categorical set of features defined as $F_{position}\left(I_{i}\right)$. These categories reflect equally sized regions of the spectrum based on the mass of the current peak relative to the parent ion mass (*pI*). For example, if we assume the number of regions *n *= 5, the lowest mass peak in the spectrum would have the feature value $F_{position}\left(I_{0}\right)=\left\langle 1,0,0,0,0\right\rangle ,$ and the highest mass peak would have the value $F_{position}\left(I_{k}\right)=\left\langle 0,0,0,0,1\right\rangle$. The relative cleavage position features capture the variation in peak intensity across the mass range of the instrument. Typically, peaks tend to be more intense near the center of the peptide and less intense or missing near the terminal ends. Fragmentation characteristics can also vary based on the relative cleavage position. The input layer of the neural network has a node corresponding to each of the categorical features, *c *= 0, 1, *..., n - *1, which are assigned either 0 or 1 as follows:

$$F_{position}{\left(I_{i}\right)}_{c}=\left\{\begin{array}{cc} 1 & if\frac{c}{n}\leq x_{i}/pI<\frac{c+1}{n}\\ 0 & \text{otherwise} \end{array}\right)$$

where *x_i_* is the mass of *I_i_*, and *pI *is the mass of the parent ion.

When a cleavage occurs between two amino acids, there are several other peaks that are observed with high frequency. These peaks correspond to isotopologues, neutral losses, doubly charged ions, and complementary ions. The remaining features use relative intensity and mass offsets to compute feature values. Given the current peak *I_i_*, an expected offset *δ*, and a mass tolerance ε; the offset peak *I_j _*is the maximum intensity peak in the range [*x_i _*+ *δ *- ε, *x_i _*+ *δ *+ ε]. The experimental offset is then defined as *δ' *= *x_i _- x_j _*+ *δ*. For a given offset peak *I_j _*the relative intensity is defined as $y_{j}^{\prime }=y_{j}/y_{i}$.

**Figure 2:**
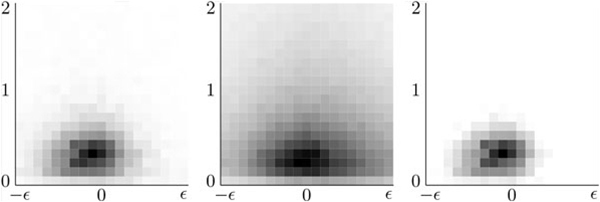
**Isotopologue histograms**. (a) The experimental mass offset (*δ'*) and relative intensity (*y'*) for the first isotopologue of a *b*-/*y*-ion. (b) The experimental mass offset and relative intensity for the first isotopologue of an unknown ion (not *b*-/*y*-ion). (c) The log-odds ration between (a) and (b)

The principal isotope feature is taken from a two dimensional histogram that models the relative intensity and mass offset of the first isotopologue. This histogram, shown in Figure 2, was computed by summing the frequency of mass offset and relative intensity within bins of size 0.05 and 0.1, respectively, for *b*-/*y*-ions in the dataset. The presence of a lower intensity isotopologue at offset *δ *= 1 serves as positive evidence that the current peak is a *b*-/*y*-ion. This can be demonstrated by building a histogram for peaks that are labeled *u*-ions, and then computing the log-odds ratio between these two distributions, as shown in Figure 2. The principal isotope feature value is sampled from this histogram based on the *δ' *and *y' *values of a candidate isotopologue of *I_i_*. The isotopologue feature indicates the current peak is an isotopologue of the peak at offset -1 Da. If the current peak has been labeled as an isotopologue, then the isotopologue feature value will be 1, and 0 otherwise. Adding this feature increases precision without affecting recall. This is due to the observation that, if a peak's isotopologue feature is 1, it will most likely be classified as a *u*-ion by the neural network.

If the current peak is a *b*-ion then we will often see the complimentary *y*-ion peak, and likewise for the converse. It is tempting to use a 2D histogram to model this feature. However performance degrades if we consider relative intensity since we do not want to penalize a candidate complementary peak for having a non-average relative intensity. The complement feature in the *net*_1 _pattern is taken from a normal distribution centered at the offset where a complementary ion is expected to be if the current peak is a *b*-/*y*-ion. By constructing a one dimensional histogram of the complementary ion mass offset, it was observed that the offset frequency is approximately Gaussian and can be modeled as X~N(0,0.1), where the 0 mean is centered around the expected complementary ion mass *x_j _*= *pI - x_i _*+ 1. The feature value is then defined as

$$F_{complement}\left(I_{i}\right)=X\left(\delta^{c}\right)$$

where *δ^c ^*is the difference between the expected and the experimental complementary ion mass. In the case of the *net*_2 _pattern, the complement feature value is the maximum *b*-/*y*-ion probability estimate produced by *net*_1 _for any peak found at the expected complement mass offset.

The H_2_O, NH_3_, H_2_O-H_2_O, H_2_O-NH_3_, and CO neutral loss features are computed by summing the relative intensity and the Gaussian estimate of the offset frequency, as described above. For example, given the neutral loss peak *I_j _*at the offset *δ *= -18.015, the feature value is defined as

$$F_{-{\text{H}}_{2}\text{O}}\left(I_{i}\right)=y_{j}^{\prime }+X\left(\delta^{\prime }\right)$$

The N-term and C-term flanking ion features are part of the *net*_2 _feature vector that rely on *b*-/*y*-ion probability estimates computed by *net*_1_. To compute the N-terminal flanking residue feature we take the maximum *b*-/*y*-ion probability, as estimated by *net*_1_, of any peak with a negative mass offset (lower mass) from the current peak that is equivalent to the mass of one of the twenty amino acids *±*0.25 Da. The C-terminal flanking residue feature is computed similarly but with a positive mass offset (higher mass). Note that the feature value corresponds to the flanking peak's *net*_1 _probability estimate, not the mass difference, and therefore does not capture any sequence information.

## Results

**Figure 3:**
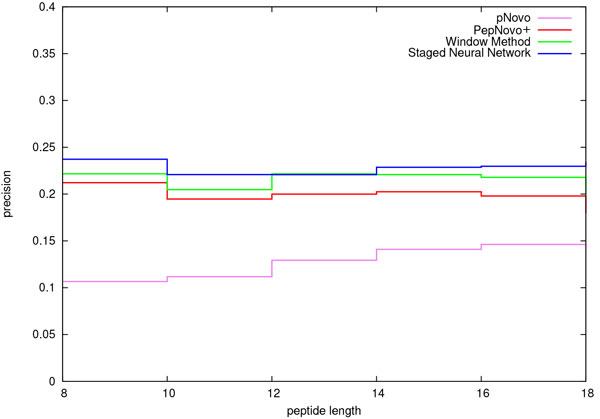
**Precision**. Cross validation comparison of precision in *b*-/*y*-ion identification for the PNNL data set. The parameter setting for the ms2preproc window method was *X *= 3, *Y *= 0, *Z *= 56.

**Figure 4:**
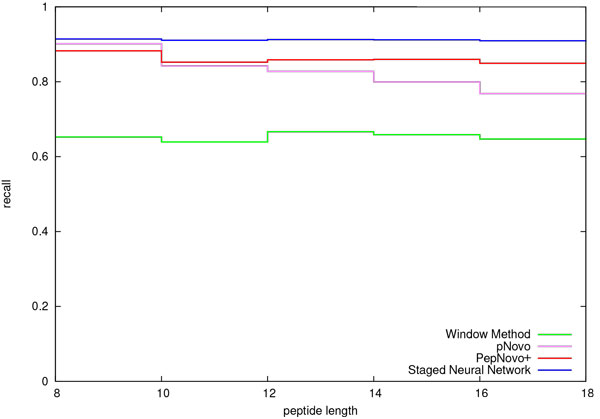
**Recall**. Cross validation comparison of recall in *b*-/*y*-ion identification for the PNNL data set. The parameter setting for the ms2preproc window method was *X *= 3, *Y *= 0, *Z *= 56.

We compared the performance of the staged neural network (SNN) peak selection method with that of two other well-known de novo peptide sequencing algorithms: PepNovo+ and pNovo. We evaluated the accuracy of the staged neural network (SNN) in correctly identifying informative peaks and compared it to that of PepNovo+ and pNovo. The results of this comparison are shown in Figures 3 and 4. Figure 3 shows the precision attained by the competing meathods. Figure 4 shows the corresponding recall. In addition, we included the window method described by Frank in the early PepNovo paper [1]. In the case of the window method, a sliding window of 56 Da is used. Within the sliding window, the three largest peaks are selected. For the purpose of comparison, we implemented a version of the window method using ms2preproc. As is evident in Figure 4, the performance of PepNovo+ is substantially better than that of the window method. Note: since PepNovo+ does not directly output the peaks that it selects for subsequent construction of the spectrum graph, we modified the source code in order to output these peaks.

Figure 3 shows that the SNN method outperforms the precision of the window method for all peptide lengths except length 12, in which case there is a tie. In this figure we also see that the precision of the SNN method is consistently greater than that of PepNovo+ and pNovo for all peptide lengths in this data set. This is a significant result since it follows that the number of retained peaks directly impacts the size of the candidate space. While precision is very important, recall is no less important. The results of recall comparison are shown in Figure 4. The recall of the SNN method is higher than that of all of the other approaches for all peptide lengths in this data set.

**Figure 5:**
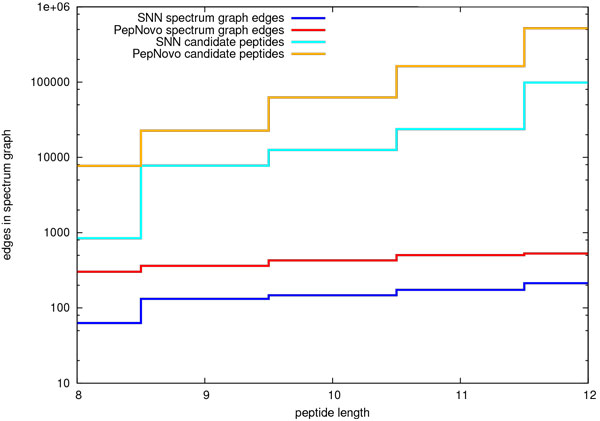
**Effect on candidate peptide search space**. The top pair of curves compares the median number of candidate peptides enumerated for a given peptide length. The bottom pair of curves compares the median number of edges in a spectrum graph for a peptide of a given length. This figure demonstrates the exponential relation between the number of edges in the spectrum graph and the number of candidate peptides.

Figure 5, which depicts the peptide candidate space, demonstrates the relation between peaks/edges and candidate search space. In order to get an estimate of the search space implied by the peaks selected by PepNovo+ and SNN, we programmed a simple candidate generating algorithm based on the method of Lu and Chen [11]. As can be seen in Figure 5, the candidate space is exponential in the size of the spectrum graph. Thus Figure 5 is limited to peptides of maximal length 12. We caution the reader to bear in mind that PepNovo+ and other de novo programs are more efficient in searching the candidate space and take a more sophisticated approach to generating candidate peptides, avoiding an exhaustive search. Nonetheless, this comparison depicts the extent of the basic candidate space implied by the selected peaks. It demonstrates that the extent of the implied SNN search space is smaller than that of PepNovo+.

**Figure 6:**
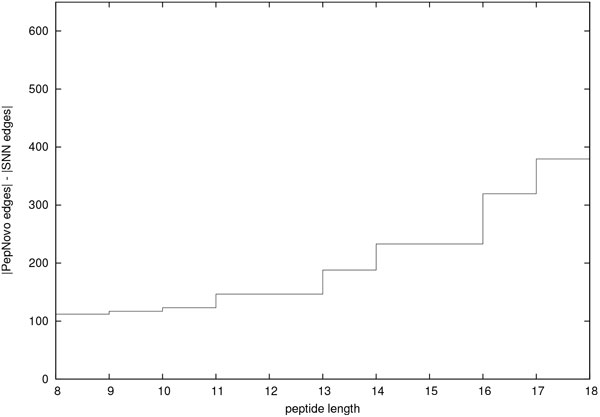
**Reduction in graph edges**. Spectrum graphs based on peaks selected by the SNN method have consistently fewer edges that do those for PepNovo+. The curve was created by taking the difference in the median number of edges in corresponding spectrum graphs grouped in bins of width 2.

As noted above, the extent of the search space is exponentially proportional to the size of the spectrum graph. We therefore use the number of selected edges in the spectrum graph as a representative measure of candidate space. This allows us to extend the comparison between SNN and PepNovo+ to longer peptides without having to exhaustively generate the implied candidates. Figure 6 depicts this comparison between the search space of SNN and PepNovo+ for peptides up to length 18. In this figure we see that the *b*-/*y*-ion recall of the SNN method is superior to that of PepNovo+ for all peptide lengths in this data set. Consequently, the best candidates from the SNN candidate space could reasonably be expected to be superior to those of the PepNovo+ candidate space. Analysis of the results depicted in Figure 6 indicate that on average there are approximately 200 fewer edges in SNN spectrum graphs than those of the corresponding PepNovo+ spectrum graphs.

The results depicted in Figures 3, 4, 5, and 6 are consistent with those that were generated from a smaller data set [6]. That data set from PNNL consisted of 3373 spectra of unique peptides from Salmonella Typhimurium. The peptides that we selected from the PNNL data set had a mass that ranged from 600 to 3000 Da and an Xcorr score of at least 2.5. We may conclude that together these two sets of results accurately depict the relative improvement in performance of the SNN peak selection method over that of PepNovo+ and pNovo.

## Discussion and conclusion

De novo peptide sequencing addresses the ill-posed problem of deriving the correct peptide sequence corresponding to a tandem MS spectrum. The first critical step in this problem is that of identifying those peaks representing *b*-/*y*-ions. It is of great importance that spectral peaks be filtered as agressively as possible while retaining informative peaks. Accomplishing this goal results in two very practical benefits: First, the smaller spectrum graphs that result from reducing the number of peaks under consideration allow spectra to be processed at a faster rate. Second, reducing the number of peaks makes it possible to process larger peptides. As discussed in the previous section, the SNN method consistently selects fewer peaks than do PepNovo+ and pNovo. The corresponding candidate search space is significantly smaller. SNN spectrum graphs on average contain 200 fewer edges than do corresponding PepNovo+ spectrum graphs. A comparison of candidate search spaces shows that the median SNN search space is consistently smaller than that of PepNovo+. As demonstrated in an earlier publication, the median SNN candidate space for peptides of 20 residues is smaller than that of PepNovo+'s median space for 15 residues [6]. In principle, all other things being equal, one could expect to be able to process longer peptides with the SNN approach. As important as it is to reduce the number of peaks under consideration, it is just as important to retain those peaks representing actual *b*-/*y*-ions. Otherwise, the resulting peptide candidate search space may not contain the correct peptide. In the preceding section we presented results that demonstrate that the SNN peak selection accuracy exceeds that of pNovo and PepNovo+ for all peptide lengths represented in the data set (Figures 3 and 4). On average, the SNN method selects significanly fewer peaks than do pNovo and PepNovo+. At the same time, on average the SNN method retains more peaks that correspond to *b*-/*y*-ions than do pNovo and PepNovo+.

The Staged Neural Network method for peak selection exhibits an improvement of accuracy in *b*-/*y*-ion peak identification over current state-of-the-art approaches. This results in a reduced, better focused candidate space. In turn, this will allow de novo sequencing programs to evaluate spectra more rapidly as well as handle longer peptides.

## Authors' contributions

JC designed and implemented the software, assisted in the design of experiments, and carried out the experiments. JR assisted in the design of the software as well as the design of the experiments and the analysis of the results. JC wrote the manuscript and JR edited it. Both authors read and approved the manuscript.

## Competing interests

The authors declare that they have no competing interests.

## Declarations

The publication costs for this article were funded by the corresponding author.

This article has been published as part of *Proteome Science *Volume 11 Supplement 1, 2013: Selected articles from the IEEE International Conference on Bioinformatics and Biomedicine 2012: Proteome Science. The full contents of the supplement are available online at http://www.proteomesci.com/supplements/11/S1.
